# Growth and formaldehyde degradation of photoheterotrophic *Methylobacterium* within radiation fogs

**DOI:** 10.1128/mbio.00463-26

**Published:** 2026-05-11

**Authors:** Thi Thuong Thuong Cao, Pierre Herckes, Derek Straub, Soumyadev Sarkar, Ferran Garcia-Pichel

**Affiliations:** 1 School of Molecular Sciences, Arizona State University 7864, Tempe, Arizona, USA; 2 Center for Fundamental and Applied Microbiomics, Biodesign Institute, Arizona State University 7864, Tempe, Arizona, USA; 3 Department of Earth and Environmental Sciences, Susquehanna University 7296, Selinsgrove, Pennsylvania, USA; 4 School of Life Sciences, Arizona State University 7864, Tempe, Arizona, USA; The University of Oklahoma, Norman, Oklahoma, USA

**Keywords:** photoheterotrophs, aerobiome, bioaerosol, bacterial diversity, *Methylobacterium*, C1 metabolism

## Abstract

The atmosphere contains thousands to millions of bacterial cells per cubic meter. However, it remains unclear if microbes are at all active or growing *in situ* or whether they are merely being transported in an inactive state. Based on the analyses of 32 overland radiation fog events over a 2-year period, we show that fog waters, with bacterial concentrations similar to those in continental or marine bodies of water, contain microbiomes well differentiated in composition from those in the dry aerosol microbiomes that occur locally before, during, or after fog events. They are consistently and strongly enriched in photoheterotrophic *Methylobacterium* species, suggesting that fog populations may be metabolizing volatile C1 compounds *in situ*, although phototrophy seems much less important. Indeed, metabolically active bacteria in the fog, and representative isolates of the main field populations, can degrade formaldehyde at unprecedently high rates; most of this activity seems to play a detoxification role. The increase in bacterial aerobiome counts upon intervening fog events, the dependence of microbial concentration on ambient temperature, the increases in cell size and frequency of dividing cells in fog water with respect to cells in interstitial aerosols of fogs, in addition to their metabolic capacity, all suggest that the fog water microbiome is actually growing. Consequently, droplets of atmospheric water should be considered a potential aquatic microhabitat. Our results highlight the fog microbiome’s role in atmospheric chemistry and have implications for fog harvesting as a source of fresh water for human use.

**IMPORTANCE:**

While bacteria are common in the atmosphere, their activity *in situ* has remained unclear. Using stagnant radiation fogs as new study systems where sampling is optimal, the dynamics, composition, cellular characteristics, and metabolic rates of fog water microbiomes, dominated by *Methylobacterium* sp., show that they are a hub of active detoxification of atmospheric formaldehyde and likely growing *in situ* on the basis of heterotrophic or photoheterotrophic metabolism of volatile C1 compounds, with implications for atmospheric chemistry and fog harvesting as sources of freshwater.

## INTRODUCTION

Bacterial activity in the aerobiome can only occur when bacteria are fully hydrated in liquid atmospheric droplets, primarily as fog or clouds. Some evidence suggests that bacteria could potentially be metabolically active in aerosol particles (1), although applicability to the atmosphere has yet to be shown directly. Hence, it is yet unclear if bacterial activity, or even growth, in clouds and fogs could determine the overall internal growth dynamics and functional roles of the aerobiome, and whether the atmosphere should be viewed as a microbial habitat rather than just as a vector for microbial dispersal. Atmospheric droplets do contain diverse bacterial communities (2–4) with viable bacteria that can be cultured (5–7). At least some such cultures seem adapted to the atmosphere’s environmental extremes (8). Furthermore, comparisons of clouds or fog with local, clear air sometimes show a biomass enrichment (4). A compositional difference between aerosols and liquid droplet fractions of the aerobiome has been detected in some instances (4), but not in others (3). This type of comparative approach suffers from the lack of certainty that one is sampling the same air parcel (i.e., lack of Lagrangian sampling). The most convincing evidence for bacterial activity in clouds comes from tracer incubations of collected cloud water, yielding heterotrophy rates comparable to those of lake water or seawater bacteria (9), although the nucleoside substrates used as tracers are not present in the air.

To gain insights into the role of atmospheric droplets as microbial microhabitats, we conducted an experimental campaign encompassing 32 radiation fog events over central Pennsylvania spanning 2 years. Unlike advection fogs or clouds, radiation fogs form locally in stagnant air masses, providing an advantage to detect true differences in time that may better reflect internal aerobiome dynamics, but this type of fog has yet to be studied microbiologically. We determined bacterial composition, the influence of fog formation on aerobiome dynamics, and the aerobiome partitioning between droplets and interstitial aerosol particles. We also determined microbial metabolic activity and growth potential of droplet bacteria, seeking confirmatory evidence in studies of pedigreed bacterial isolates representative of major native populations found in the fog water microbiome. This provided not only a first look at the radiation fog microbiome but also evidence that fog droplets indeed constitute a microbial microhabitat of key relevance for atmospheric chemistry.

## RESULTS

### A unique, dense fog water microbiome

**Fig 1:**
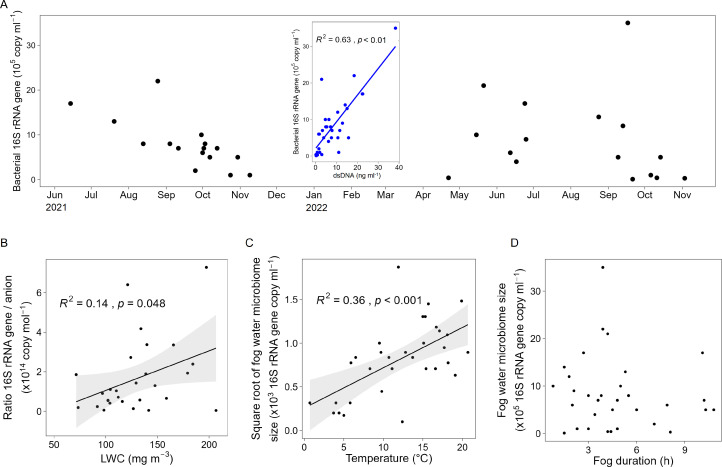
Characteristics and trends of the fog water microbiome across 32 sequential radiation fog events. (**A**) Concentration of bacterial 16S rRNA gene copy number. The inset shows the correlation between 16S rRNA copy number and total extractable double-stranded DNA (dsDNA). In most events, dsDNA content detected in the blank water was less than 1.0 ng mL^−1^ (Data set S1). (**B**) The ratio of bacteria to anionic solutes in fog water correlates positively with liquid water content (LWC) in the air; relationships between LWC and bacteria and LWC and anionic solutes are presented separately (Fig. S1). (**C**) The square root of bacterial concentration in fog water correlates linearly with ambient temperature. The shaded area represents the 95% CI of the linear regression. (**D**) Bacterial concentration in fog water does not show a dependency on the duration of the fog event. See text for statistics.

**Fig 2:**
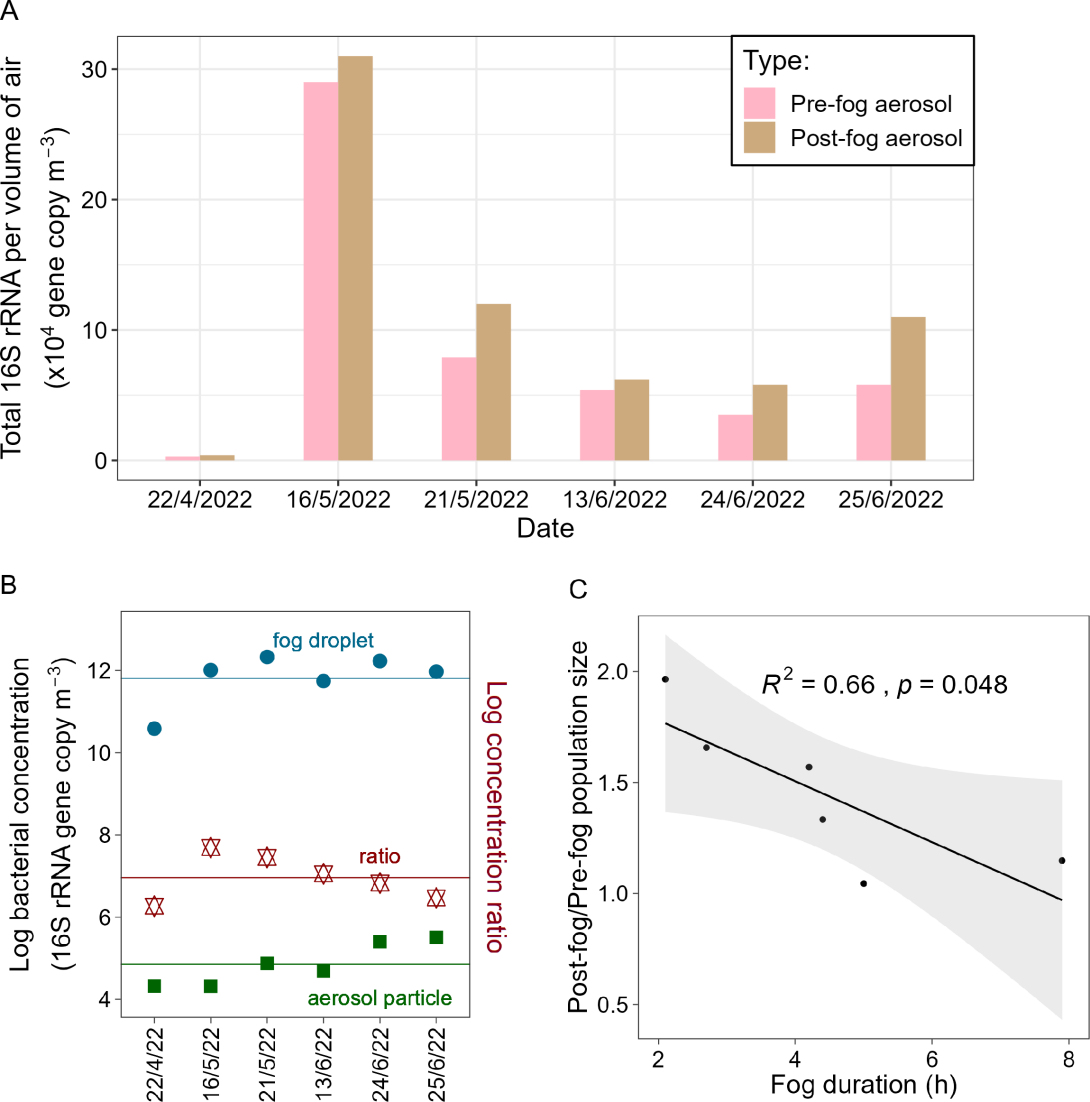
Changes in aerobiome size after, and partition into liquid and interstitial aerosol during, fog events. (**A**) Size of the bacterial population in the air before and after six radiation fog events. (**B**) Partition of bacterial populations into fog droplets and interstitial aerosol particles during fog events, all expressed as volumetric concentration in each fluid. Concentration data are mean values of three technical replicates, and SD was omitted for clarity (always <1% of mean); horizontal lines are averages among events. (**C**) Ratio of bacterial population size before and after a fog event as a function of fog event duration. The shaded area represents the 95% CI of the linear regression.

The fog water microbiome was substantial as gauged by its 16S rRNA gene content, averaging around 10^6^ copies per mL of liquid water, although also quite variable among events (Fig. 1A). Total biological loads in droplets, assessed by total extractable DNA concentration, gave consistent results (Fig. 1A, insert), indicating that bacteria drove total biological load in fog water. We confirmed that conditions were stagnant during sampling, allowing for Lagrangian sampling during each event (Data set S1). In a subset of six events, we sampled concurrently droplets and interstitial aerosol particles during fog. From these paired data, fog water populations were 6–7 orders of magnitude more concentrated than populations in the dry particle fraction. However, water droplet and interstitial aerosol microbiomes within a given volume of foggy air tended to be commensurate [*n* = 6, *t* (5) = 0.43, *P* = 0.76] because fog water droplets occupy only a very small fraction (1.3 × 10^−5^ percent) of this volume (Data set S2; Fig. 2B). Fog droplets thus represent a major, concentrated bacterial biomass hub in the air during fog, with concentrations within the range found in eutrophic lake (10) and ocean waters (11). Bacterial concentrations in fog droplets tended to increase with the fog’s liquid water content (LWC, Fig. S1A; although a regression gave *R*^2^ = 0.02, *P* = 0.49 for the full data set, and *R*^2^ = 0.14, *P* = 0.09, removing two outliers). This trend is opposite to that between aqueous ionic content and LWC (Fig. S1B; a power regression yielding *R*^2^ = 0.12, *P* = 0.07). Combining these two parameters, the ratio of biological vs. chemical contents in fog water was proportional to its LWC (Fig. 1B; *R*^2^ = 0.14, *P* = 0.05, *n* = 28). Hence, fundamentally different processes that result in opposite trends with LWC must control the concentrations of bacteria vs. inorganic solutes in fog droplets, where bacterial droplet concentration cannot be explained by simple partitioning of the bacterial concentration in the gas phase, as the inorganic solutes can. Bacterial concentrations in droplets tended to increase with ambient temperatures, the square root of bacterial concentration being linearly related to temperature (Fig. 1C; *R*^2^ = 0.36, *P* = 3 ⨯ 10^−4^ for the full data set), following the relationship of bacterial growth rate with temperature (12). These two relationships are consistent with *in situ* growth playing a role in the concentration of the fog water microbiome. However, bacterial numbers seemed independent of fog event duration (Fig. 1D). Hence, while bacterial growth may partly explain the size of the fog water microbiome, other factors, such as net losses through wet deposition, likely also play a role. Because the bacterial gains (defined as 16S rRNA copies after vs. before) across a fog event were inversely proportional to its duration (Fig. 2C), time-dependent losses must have occurred, which is consistent with a continuous process of droplet deposition.

**Fig 3:**
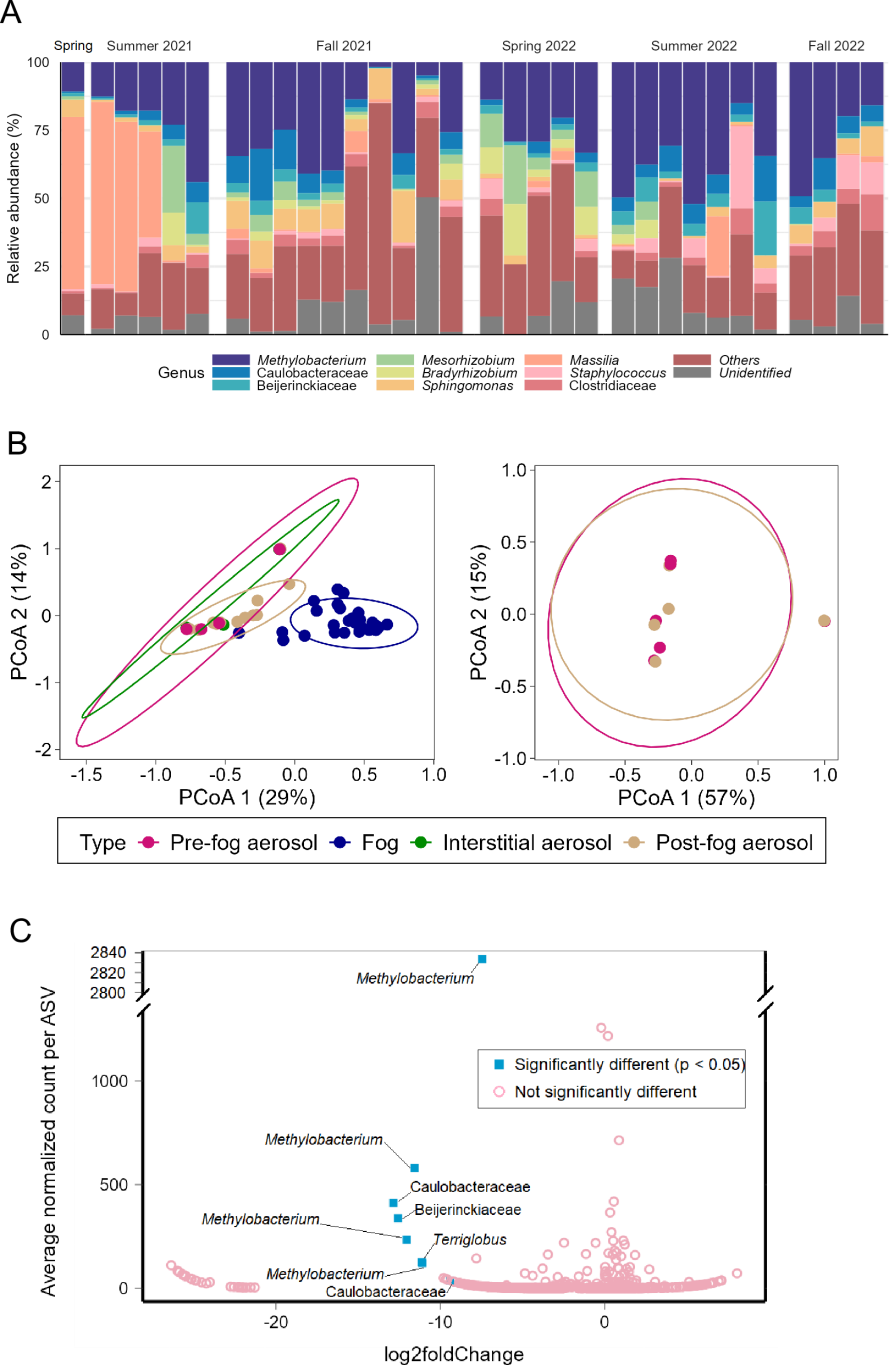
Compositional analyses of the fog microbiome and partition of the bulk aerobiome. (**A**) Fog water bacterial community composition in 32 sequential fogs was determined by 16S ribosomal gene sequencing with phylogenetic assignments to the level of genus according to the SILVA database. (**B**) Fog water and aerosol microbiomes differ in community composition. The left panel shows a principal coordinates analysis (PCoA) separation by microbiome type in the full set of data based on Bray-Curtis dissimilarity. The right panel shows the subset of paired data for post- and pre-aerosol microbiomes (six events). Ellipses, color-coded to match, encompass 95% confidence areas. Parallel analyses using the Jaccard dissimilarity index are shown in Fig. S5. (**C**) Microbial ASV partition of the aerobiome into aerosol and liquid phases during fog, as differential abundance. Solid squares indicate ASVs that were differentially abundant (*P* < 0.05), open circles denote those with non-significant preference. Negative values indicate enrichment in the fog water and vice versa. Interesting ASVs are labeled with their taxonomic assignment to the highest resolution possible.

We detected an average of 188 unique amplicon sequence variants (ASVs) per event, although this was also quite variable (SD = 106 ASVs; Data set S1). At the phylum level (Fig. S2), Proteobacteria tended to dominate most fog water microbiomes with 65% ± 18% of reads, followed by Firmicutes (12% ± 12%) and Bacteroidota (7% ± 11%). At the genus level (Fig. 3A), bacteria in the *Methylobacterium* group were consistently dominant, contributing 29% ± 13% of all reads, followed by *Massilia* (9% ± 20%), an uncultured clade of Caulobacteraceae (6% ± 5%), and *Sphingomonas* (5% ± 4%). The representation of those secondary bacterial taxa was much less consistent across events than that of *Methylobacterium*-like phylotypes (Fig. 3A). *Methylobacterium* are heterotrophs that specialize in C1 compounds, except methane (13), with some also growing as photoheterotrophs (14). Consistently, a *Methylobacterium*-specific viable count on agar media yielded 2,620 c.f.u. per mL of fog water, in which all colonies had the pink phenotype typical of *Methylobacterium* (Fig. S3)*,* an assignment confirmed in four randomly selected colonies by 16S rRNA sequencing.

### Effects of intervening fog events on the airborne microbiome

We also determined the dynamic effects of fog formation during the six events with paired data by comparing bacterial content in air before and after such events (Fig. 2A). In each case, the post-fog air contained significantly more bacteria than its respective pre-fog air (Wilcoxon signed-rank, *n* = 6, *P* = 0.02), amounting to a 45% increase on average and varying between 7% and 90%. These recurrent dynamics speak for a consistent burst in the aerobiome population size as a consequence of fog condensation, an effect that is hard to explain without invoking *in situ* growth, in the virtual absence of air movement (Data set S2). Consistently, there were increases in post-fog absolute abundance of the *Methylobacterium* ASVs that were dominant in the intervening fog water microbiomes (Wilcoxon signed-rank test, *n* = 6, *P* = 0.02). However, intervening fog events did not leave a statistically significant imprint on the relative composition of the post-fog airborne microbiome (Fig. 3B), since *Methylobacterium* remained a minor component after fogs.

### Selective partitioning of the aerobiome between fog droplets and interstitial aerosols

In the six fog events with paired determinations of all microbiome types, indices of microbial diversity were similar among them (Fig. S4), but, despite variations in composition among fog events (Fig. 3A), fog water microbiomes tended to be self-similar and well differentiated from the microbiomes existing in the air prior to fog condensation (Fig. 3B). Fog water microbiomes also differed compositionally from the interstitial aerosol microbiomes during fog events themselves (Fig. 3B). The Bray-Curtis dissimilarity algorithms used for community comparisons in Fig. 3B factor in differences in relative abundance, whereas those using Jaccard distances (Fig. S5) do not. That water and aerosol communities could no longer be distinguished from one another in the latter indicates that fog water microbiomes were likely sourced in the aerosol microbiome, differentiating by preferential enrichment of particular ASVs, rather than by incorporation of new types. Fog water microbiomes were also compositionally different from those in fog-free air after a fog event came to an end. It appears that the selectivity in partitioning does not leave a significant imprint on the aerobiome composition after fog dissipation. A detailed comparison of microbial composition between fog water and interstitial aerosol samples during foggy conditions revealed which microorganisms were preferentially enriched in each phase (Fig. S6). Preference for water droplets involved ASVs assignable to *Methylobacterium*, the Caulobacteraceae, and the Beijerinckaceae, whereas others like *Bradyrhizobium* appear to be preferentially found as interstitial dry aerosol particles. DESeq2 analyses on cumulative data show that only 7 among 1,320 ASVs detected were differentially enriched (*P* < 0.05) in droplets (Fig. 3C). Among those, the *Methylobacterium* ASVs were by far the most abundant (14%–50% of total reads in fog water, but only up to 0.3% in interstitial aerosol particles). No ASVs reached significant preferential enrichment in interstitial aerosols after discarding ASVs that were also found in the blank controls, likely of human origin, like those assignable to *Prevotella*.

### Differential cellular traits in the droplet and interstitial aerosol microbiomes

**Fig 4:**
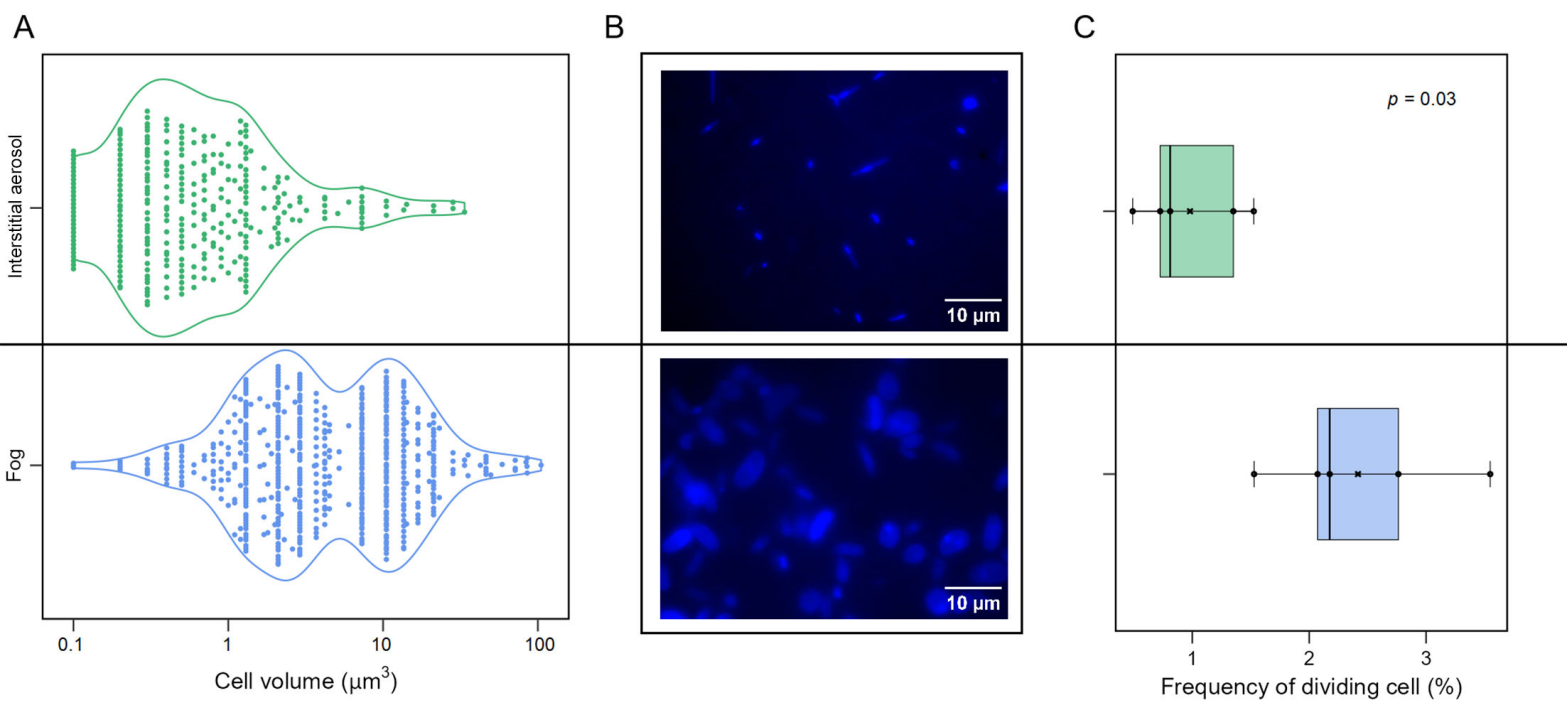
Comparative cellular characteristics of bacterial populations in interstitial aerosol particles (top panels) vs. fog water (bottom panels) during fog events. (**A**) Cell volume distribution from image analyses of microscopic images (*n* = 5 events). A parallel analysis using particle size measurements through flow cytometry is in Fig. S7. (**B**) Epifluorescence photomicrographs of DNA-stained cells, showing conspicuous size differences. (**C**) Frequency of dividing cells in the respective populations (*n* = 5). *P* values given are for fog water vs. interstitial aerosol particle populations.

To further probe the possibility of active growth in the fog water, we examined the bacterial populations directly by epifluorescence microscopy and flow cytometry. As shown in the size distributions of Fig. 4A, bacteria present in droplets were significantly larger than those in aerosol (8.5 ± 1.2 vs. 1.6 ± 3.8 µm^3^; Wilcoxon rank-sum test, *P* < 2 × 10^−16^), both measured in a hydrated state. The same trend could be observed independently through automated particle sizing by flow cytometry (Fig. S7). Droplet bacteria are larger than average, since two-thirds of all bacterial species are between 0.1 and 2.1 µm^3^ (15). This again could be consistent with active growth, since growing cells are larger than their non-growing counterparts (16), although it could also be a spurious result of the differential community composition. The strongest evidence for *in situ* growth comes from frequency of dividing cells (FDC) determinations, a standard approach in various aquatic habitats (17–19). The FDC in droplets (Fig. 4C) ranged from 1.5% to 3.5%, averaging 2.4%, which in aquatic microbiomes corresponds to doubling times of some 60 h (20). In interstitial aerosol particles during fogs, the range was from 0.5% to 1.4%, with an average of 1.0%. The two frequencies were different according to a Wilcoxon rank-sum test (*P* = 0.03). The FDC of interstitial aerosol bacteria, likely dormant in a desiccated state, only reflects the cell cycle stage at which they originally dried out.

### Metabolic basis of the fog water microbiome

**Fig 5:**
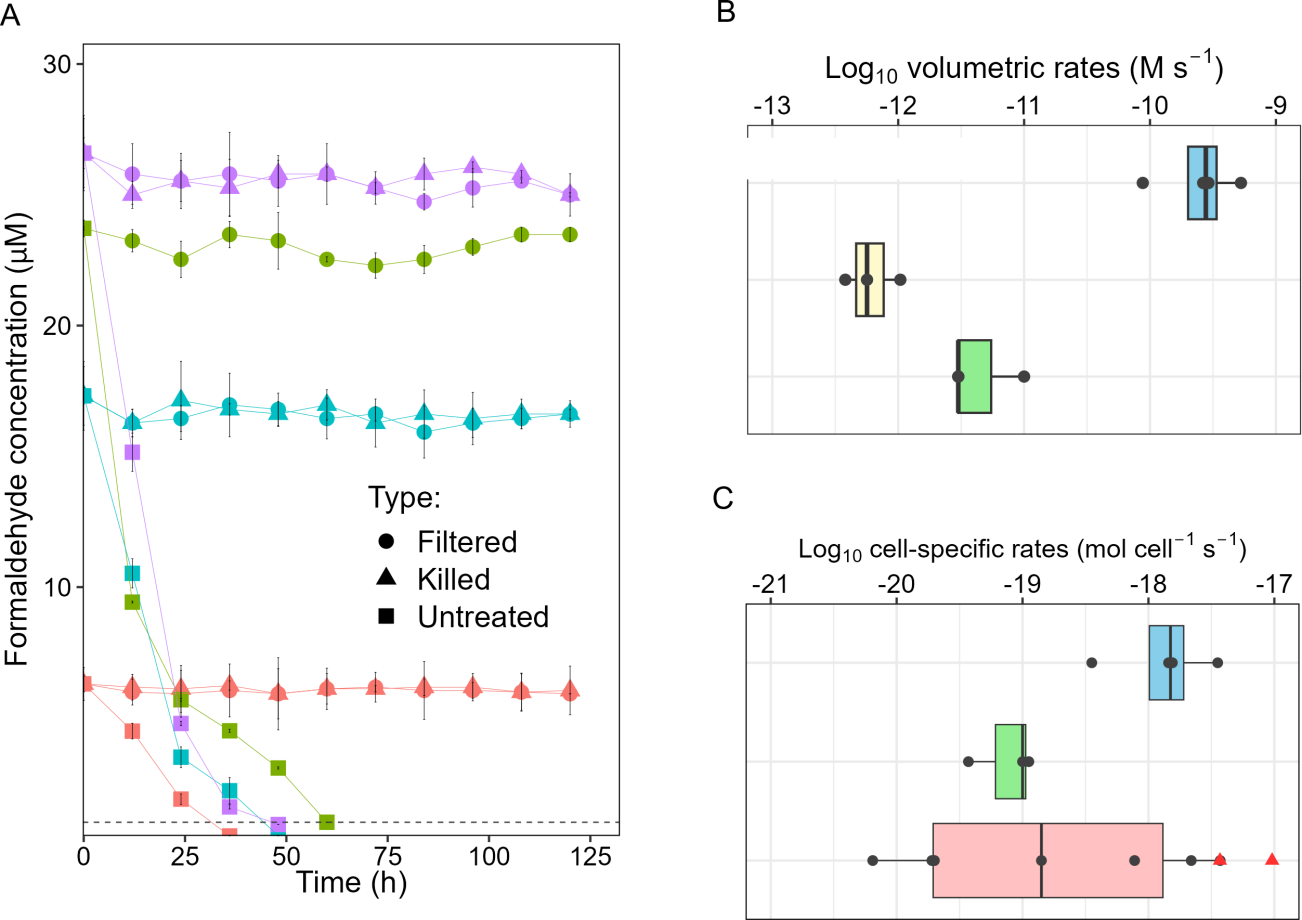
Depletion of naturally occurring formaldehyde in radiation fog water. Incubations were of freshly collected fog water in the dark at 10°C, with ambient levels of formaldehyde. (**A**) Dynamics of depletion in untreated, particulate-free, and chloroform-killed fog water. Dashed line is the analytical detection limit. Different colors show different fog events. (**B**) Pseudo-first order rates from data in panel **A** (*n* = 4), compared to literature values from cloud water (*n* = 3) (27). (**C**) Cell-specific rates, compared to literature values, and to rates obtained in pure cultures of bacteria isolated from the aerobiome (28, 29), where red dots are optimal rates for our strains (Table S2). Dots on the boxplots represent data, bars are quartiles 1 and 4, the box indicates quartiles 2 and 3, and the vertical line is the median.

Considering fog as a (micro)habitat brings forth the question of what type of metabolism might support it. A trait-based analysis of the ASVs common in fog water (Fig. 3A) offers guidance. Since none could be assigned to known autotrophs, the basis must be heterotrophy. The fact that no phylotypes fit known lithotrophs suggests an organotrophic system. As sources of energy and carbon, bacteria in droplets can principally count on an ample supply of volatile organic compounds (e.g., alcohols, aldehydes, and organic acids [21]), which, depending on their solubilities, will also equilibrate into fog droplets. The recurrent dominance of *Methylobacterium* suggests that the fog microbiome may be crucially based on the oxidation of volatile C1 organics and may possibly benefit from phototrophy. To test the former, we followed the removal of formaldehyde, a common (and toxic) volatile in the atmosphere (22), through incubations of freshly collected fog water. Formaldehyde, present at concentrations between 6 and 25 µM in our samples, can reach up to several hundred micromolar (23–25). Existing formaldehyde at the start of the incubation was swiftly consumed to undetectable levels (Fig. 5A), with rates around 2.9 ± 1.8 × 10^−10^ M s^−1^ (or some 25 µM day^−1^), roughly 200-fold faster than rates measured elsewhere in cloud water (26) (Fig. 5B). While there are uncertainties in the rate measured stemming from assaying the samples as bulk liquid rather than droplets, any such effects must have been present in the literature determinations used here for comparison. On a per cell basis, derived using microscopic cell counts in the incubation water (Table S1), this corresponds to 2.3 ± 1.4 × 10^−18^ mol cell^−1^ s^−1^, 30-fold faster than measured in cloud water (27), and close to the maximal rates measured in the cultures of bacteria (28, 29) (Fig. 5C). Most importantly, the activity was associated with the particulate fraction in the fog water samples, as it could be virtually halted by filtration, and our controls using chloroform-killed fog water clearly showed that most of it (95% on average) could be ascribed to biological processes. If the extant bacterial population was oxidizing formaldehyde solely to support growth with growth efficiencies typical of cultured methylotrophs = (around 8 g [dry mass] per mol of substrate [30]), they could theoretically be doubling biomass in a few minutes, unreasonably fast for any known microbe. Hence, biological formaldehyde degradation must largely serve energy generation or mere detoxification purposes (31), rather than exclusively supporting growth. To address the potential role for photoheterotrophy, we examined the cumulative fractional time during which fog was in the dark over our sampling campaign. Most of the fog occurred at night, but not all (Fig. S8). Defining nighttime by visible light intensities below 3.4 Wm^−2^, on average (*n* = 34), fogs were in the dark 60% of the time.

### Activities and traits in representative isolates of dominant fog water populations

We also isolated *Methylobacterium* sp. strains from our fog waters, from which two strains were selected for further inquiry in the laboratory. Strain SUH_01 and strain SUH_02 were chosen as their 16S rRNA sequences matched fully the two ASVs that represented the most commonly occurring and largest field populations. Complete genomes were obtained for both strains. The genome of *Methylobacterium* sp. strain SUH_01 was 6.14 Mbp in size, assembled into 129 contigs, with 6,529 open reading frames, of which 3,329 were protein-encoding genes for which a function could be assigned bioinformatically. *Methylobacterium* sp. strain SUH_02 had a genome size of 7.19 Mbp, as assembled into 419 contigs. A total of 7,420 open reading frames were identified, of which 3,922 were protein-encoding genes with functional assignments based on sequence similarity. Both strains contain a full set of homologs required for the serine cycle in their genomes, supporting their ability to assimilate formaldehyde (HCHO). Notably, SUH_01 had two copies of genes encoding enolase (EC 4.2.1.11), phosphoenolpyruvate carboxylase (EC 4.1.1.31), malate-CoA ligase (EC 6.2.1.9), and Malyl-CoA lyase (EC 4.1.3.24). Both genomes contained full sets of homologs for bacteriochlorophyll synthesis, indicating a potential for photoheterotrophy.

Relevant physiological traits determined for these strains are gathered in Table 1 and expanded in Fig. S9 and S10 and Table S2 SUH_01 cells had volumes of 3.3 µm^3^ and those of strain SUH_02 were 2.75 µm^3^.

**TABLE 1:** Compendium of physiological traits of *Methylobacterium* strains^*a*^

Parameter	SUH_01		SUH_02	
	Light	Dark	Light	Dark
Biodegradation rate (⨯10^−18^ mol cell^−1^ s^−1^)	9.8 ± 0.1	9.6 ± 0.5	3.6 ± 0.1	3.7 ± 0.3
Grow rate on HCHO (day^−1^)	0.09 ± 0.01	0.09 ± 0.00	0.07 ± 0.01	0.07 ± 0.01
Grow rate on glucose (day^−1^)	0.13 ± 0.01	0.10 ± 0.02	0.20 ± 0.01	0.10 ± 0.01
HCHO toxicity	ND	Low	ND	High

^*a*^ Details for each determination are found in Fig. S9 and 10; Table S2. For growth in the light, values obtained at 100 µmol (photon) m^−2^ s^−1^ are shown. ND, not determined.

Both strains degraded formaldehyde during week-long incubations at 1 mM or 100 µM. At 10 mM (Table S2), strain SUH_01 degraded 68% of the substrate in a week, but no depletion was observed in SUH_02. At 100 mM, neither strain showed activity (i.e., no difference from killed controls). Optimal cell-specific rates are given in Table 1, from which two conclusions can be drawn. First, the rates are equal to or slightly higher than those determined in our fog incubations, and as high as rates determined in other atmospheric isolates elsewhere (Fig. 5C), qualitatively consistent with the notion that *Methylobacterium* is responsible for the high activities measured in the field. Second, there seems to be some physiological specialization between the two isolates, SUH_01 operating optimally around 1 mM and SUH_02 around 0.1 mM or lower. We observed net growth with formaldehyde as the sole source of carbon in both strains, maximally at 1 mM formaldehyde for SUH_01 and at 200 µM for SUH_02, with doubling times around 184 and 237 h, respectively, similar to those attained with glucose as a carbon source. To test the possibility that phototrophic energy generation might enhance these rates, we measured the enhancing effect of light from incandescent bulbs (so as to optimize the excitation of BChl a through their rich IR output) on formaldehyde oxidation but found no effects. (Fig. S10). Photoheterotrophy was detectable when cells were growing on glucose (Fig. S10) and was apparently more effective in strain SUH_02, but not detectable when growing on formaldehyde. Formaldehyde exposure at high concentration, however, was toxic to both strains, as determined by viability counts on formaldehyde-free R2A medium. For SUH_01, no viable cells remained after 48 h of exposure to 100 mM, but for SUH_02, 4 h of exposure sufficed to attain the same result. With exposures at 10 mM, seven days were needed to bring SUH_01 viability down one order of magnitude, but 24 h sufficed to reduce viability beyond two orders of magnitude in SUH_02. At 1 mM, SUH_01 was essentially impervious, while 24 h reduced the viability of SUH_02 by one order of magnitude (Fig. S9). This sensitivity, even at concentrations where cells were oxidizing formaldehyde actively, speaks for the relevance of their activity as a detoxification mechanism, at which SUH_01 seems clearly superior, mirroring its higher genomic endowment for the serine pathway. Photoheterotrophy was detectable when cells were growing on glucose (Fig. S10) and apparently more effective in strain SUH_02. Thus, physiological characterization confirmed C1-based growth capacity of these strains, and likely of their populations of origin. It also confirmed their potential for photoheterotrophy, but only under conditions of light intensity and carbon source availability unlikely to be of consequence during growth *in nebula*.

## DISCUSSION

Our data show that the radiation fog water microbiome presents a unique composition compared to interstitial aerosol microbiomes during fog and also compared to clear-air microbiomes preceding or succeeding fog events, likely a consequence of preferential bacterial recruitment from aerosols. Multiple lines of evidence, including increases in the aerobiome size with intervening fog events, its dependence on temperature, the presence of larger cells, and the high frequency of dividing cells, all speak for a fog water microbiome that is also capable of growth *in nebula*. While one could find alternative explanations for each of these phenomena separately, *in situ* growth remains the most parsimonious explanation for all concurrently. That the conditions for heterotrophic activity based on C1 compounds like formaldehyde that are available in the air lead to exceptionally high biodegradation rates in the fog water microbiome is consistent with that notion. However, only a fraction of the formaldehyde processed could be used directly for growth, and most of the activity must serve as a detoxification mechanism. This is also supported by the genetic and physiological traits of cultured isolates representative of major bacterial populations in natural fog water. One should note that volatiles other than formaldehyde contribute to the repertoire potentially available to fog microbes (and these are much less toxic). Hence, our experimental conditions should be regarded as quite restrictive. The roles of other volatiles remain to be tested and could not only be a contributing factor to *in situ* growth but also to community diversity through niche diversification, as seen in other habitats like soil (32), and as suggested by the differences in our isolates. Atmospheric water in fogs should be considered a true aquatic microhabitat, if a temporary one, able to support strong activity and even growth of a dense assemblage of microorganisms recruited locally, effectively, and non-randomly from aerosols.

The dominance of *Methylobacterium* adds fogs to this microbe’s known preferred niches of plant surfaces and soil (33, 34) to which they likely return with atmospheric wet deposition, thus establishing a continuum of niches connected by physical processes of entrainment and deposition. While phototrophy does not seem to play a crucial role in its survival during nighttime radiation fogs, it may serve them well in other parts of this continuum or perhaps in the presence of a more diverse source of C compounds, as suggested by our culture experiments. The unprecedented effectiveness of fog bacteria in the biodegradation of volatile contaminants, as exemplified by formaldehyde, may thus be key for atmospheric chemistry. However, harvesting of fog water is currently held as a sustainable source of freshwater supply in some locales, often in underdeveloped countries, where it is touted by some agencies to be free from contaminants and harmful microorganisms (35). Such advice may need to be revised, given the number of bacteria detected in fog, and that some (like *Methylobacterium*) are potential opportunistic pathogens. Furthermore, actively harvesting fog water may deprive the atmosphere of a natural, local detoxification hub with unintended side effects.

## MATERIALS AND METHODS

### Fog and aerosol collection

Fog and aerosol samples were collected at Susquehanna University’s Center for Environmental Education and Research (CEER; 40.79^o^N 76.88^o^W) near Selinsgrove, PA, USA. The site is characterized by open grassy fields and is surrounded by active farmland. Fog samples were collected with an automated Caltech Heated Rod Cloudwater Collector (CHRCC) mounted 2 m above ground having a 50% cutoff particle diameter of approximately 9 μm (36). Detailed fog event detection, as well as blank and fog water collection, is described in Supplemental File. The fog collector included a downstream interstitial aerosol sample collector, so that whenever the CHRCC was activated, the droplet-free air was then filtered through 47 mm quartz fiber filters (Whatman QM-A, Cytiva, MA, USA; prebaked at 600°C for 12 h) to collect interstitial aerosol particles. These filters were housed in an Advantec MFS polypropylene, open-faced filter pack installed downstream of the CHRCC exit (Fig. S11). The flow rate through the filter pack was 22 L min^−1^ controlled by a critical orifice. A second sample collector, positioned also 2 m above ground and 2 m away from the first one, was similar in construction to the secondary sampler in the CHRCC, used identical filter packs and flow rates, and was dedicated to obtaining ambient aerosol samples for 12 continuous h before and after fog formation.

### Microscopic cell counts, cell volume, and frequency of dividing cells determination

Microscopic cell counts of natural fog samples or cultures were determined in formalin-fixed (4% final concentration) aliquots, which were DAPI (4′,6-diamidino-2-phenylindole, 0.03M, Sigma-Aldrich, MO, USA) stained for 2 min, then collected onto black 0.2 µm pore size polycarbonate membrane filters (Sterlitech, WA, USA) by filtration. The filters were then placed on microscope slides for counting using epifluorescence microscopy (Carl Zeiss AxioScope.A1) using UV excitation (380–400 nm) following (18). To assess potential bacterial growth *in situ*, the frequency of dividing cells (17) was determined on microscopic counts slides obtained as above. For interstitial aerosol samples, half of the collecting quartz fiber filter was cut and soaked in 4% formaldehyde solution for 10 min. The filter was then dried and stored at 4°C. The bacteria on the filter were then resuspended in 10 mL of phosphate buffer saline (PBS, pH = 7.0), stained with DAPI, and collected on polycarbonate filters as with the fog samples. FDC was calculated as the number of dividing cells over the total cell count. A cell was considered dividing when a cell wall constriction was visible (18). For each determination, we counted 1,500 cells in at least 10 microscopic fields. For cell volume determinations, the length and width of DAPI-stained cells were measured using ImageJ software version 1.54. Cell volume was derived from measured dimensions assuming simple formulae for cylinders. One thousand cells each were counted to generate the cell volume distribution of each sample type. The particle size distribution of paired fog and aerosol samples was measured by flow cytometry, as detailed more fully in the SI.

### DNA extraction and 16S rRNA quantification

Samples for DNA extraction were processed immediately after collection. Twenty to 100 mL of liquid fog sample was filtered through a sterile 47 mm filter funnel provided with a polyethersulfone membrane filter with a 0.2-µm diameter pore size (Pall, NY, USA), then kept at –20°C until further processing. DNA extraction used the PowerSoil DNA Extraction Kit (MoBio/QIAGEN, Germany) after the method by Mazar et al. (37) with several modifications as described in the SI. DNA concentrations in the extracts were determined via fluorometry using Qubit dsDNA high sensitivity Assay Kits (Life Technologies, CA, USA). The copy numbers of 16S rRNA genes in extracts were quantified by real-time qPCR, using the universal primers 338F 5′-ACTCCTACGGGAGGCAGCAG-3′ and 518R 5′-GTATTACCGCGGCTGCTGG-3′, performed in triplicate using PerfeCTa SYBR Green FastMix Rox (Quantabio, MA, USA) in an ABI ViiA 7 thermocycler (Applied Biosystems, MA, USA) under previously reported conditions (38). The calculated 16S rRNA gene copy number in each sample was then multiplied by the percentage of bacterial reads to attain the corrected prokaryote 16S rRNA gene copy number. The air-equivalent concentration of bacteria was defined as the 16S rRNA gene copy number obtained per unit volume of air sampled (copies m^−3^). For the fog samples, the air-equivalent concentration is equal to the cell liquid concentration (copies mL^−1^) multiplied by the liquid water content (LWC; mg m^−3^), assuming water density to be 1 g mL^−1^.

### Sequencing and bioinformatic analyses

Ribosomal RNA genes were amplified and barcoded by polymerase chain reaction (PCR) from DNA extracts using primers 515F/806R targeting the V4 region of the 16S rRNA gene. PCR products were then pooled, purified, and mixed for MiSeq sequencing following the reference method (39). High-throughput sequencing was conducted on the Illumina MiSeq platform, and resulting reads were processed with QIIME2/DADA2 for ASV generation, taxonomic assignment, and diversity analyses after read-depth normalization. Community similarity was evaluated using Bray-Curtis and Jaccard PCoA, and differential abundance was assessed with DESeq2, while contaminant ASVs were removed. More methodological details of the bioinformatic analysis are provided in the SI. For whole genome sequencing of isolated strains, genomic DNA was extracted, DNA quality checked, a library prepared, and sequencing was performed using NovaSeq X Plus Series at Novogene (Sacramento, CA, USA). Sequences were assembled using SPAdes (40). CoDing sequences (CDS) were identified by Glimmer (41), followed by Prodigal (42). The RAST tool kit used the default parameters to run the workflow (43).

### Formaldehyde degradation kinetics

For natural communities, fog water collected by a CASCC during four different events was transferred immediately to the lab for biodegradation test. A killed control was prepared by adding chloroform (Sigma-Aldrich, MO, USA) at a 1:30 ratio to the fog water. A particle-free control was also prepared by filtration through a polyethersulfone filter package with nominal porosity of 0.22 μm (Pall, NY, USA) to remove all microorganisms. Native fog water and controls were then incubated in brown, 250-mL glass bottles at 10°C (the average temperature of the fog events in the late summer and fall) in the dark for 5 days. Aliquots were collected at the beginning of the experiment and at 12-h intervals, preserved with a sulfur (IV) formaldehyde preservative solution (44) and kept at 4°C before formaldehyde determination by fluorometry (44). Biotransformation rates (M s^−1^) were calculated following the first-order loss rate as described by Husárová et al. (28). For cultures, strains were pre-grown in liquid R2 medium (45). After biomass harvesting by after 3 to 5 days of centrifugation (4,000 rpm, 15 min, 10°C), cells were washed twice in a saline solution (NaCl 0.8%) to remove residues from the R2 medium and resuspended in mineral salts medium (46), with pH adjusted to 7.0. A 16% (wt/vol), methanol-free formaldehyde solution (Thermo Fisher Scientific, MA, USA) was added to the medium to final concentrations of 100 µM, 1 mM, 10 mM, and 100 mM, as the sole C source, and the formaldehyde dynamics followed analytically as for fog water. Additionally, culture turbidity was measured by absorbance at 400 nm using a UV-2600i spectrophotometer (Shimazu, Japan) as a proxy of cell concentration and to measure growth. DAPI-based microscopic cell counts conducted as explained above were used to determine cell-specific rates (mol cell^−1^ s^−1^) for both cultures and natural fog samples.

### Viable counts, enrichment, and isolation of *Methylobacterium* from fog

Viable counts from natural fog water were conducted by plating on agar-solidified (1.5%) “artificial fog water” mineral salt medium (46) supplemented with 1 mM formaldehyde as the sole C source and pH = 6.8 with colony counting (c.f.u.) after 5 days of incubation in the dark at 10°C to mimic average fog temperatures. This yielded almost exclusively pink-colored colonies of *Methylobacter,* as determined subsequently by single-colony sequencing (see below). Viable counts were similarly conducted to determine remaining viability after exposure of cultures to formaldehyde, but these were incubated at 17°C. Bacterial strains were isolated from fog samples taken from September 2021 to October 2022. For this, 50 µL aliquots of 100-fold diluted fogwater were plated onto oligotrophic 1.5% agar R2A medium (45), amended with HCHO to a final concentration of 200 µM, and incubated at 17°C under dark, aerobic conditions. Individual colonies were picked and streaked onto R2A plates, repeating three times for each colony, transfer to obtain pure isolated strains. Genomic DNA was extracted from one colony of each isolated strain and the 16S rRNA gene amplified by PCR, using general bacterial primers 530F (5′-GTGCCAGCMGCNGCGG-3′) and 1492R (5′-GGTTACCTTGTTACGACTT-3′) (47), amplificates Sanger sequenced at the Arizona State University Genomics core facilities, and forward and reverse sequences aligned using Geneious version 8.0 (48). The resulting 800–900 bp consensus sequences were submitted to basic local alignment search BLAST (http://www.ncbi.nlm.nih.gov/BLAST/). Taxonomy was assigned based off matches (> 98% identity) to 16S rRNA sequences of strains in the NCBI database. Of the various strains obtained, two, *Methylobacterium* sp. strain SUH_01, *and Methylobacterium* sp. strain SUH_02, were selected for further work, as their 16S rRNA sequences matched fully the ASV’s of the two most commonly occurring and largest field populations in our field data set. Exponential growth rates were determined based on two-point determinations in independent incubations based on cell counts.

### Statistical analyses

All the statistical analyses were performed on the R vegan package (49). Levene’s test was used to test for equal variance, and Shapiro-Wilk test for normality. After verifying the assumptions met, analysis of variance statistical test was applied, and Tukey’s honest significant difference was used as a post-hoc test for pairwise comparisons. The Wilcoxon test was used for pairwise comparisons of data sets failing normality tests.

## SUPPLEMENTAL MATERIAL

Data set S1 mbio.00463-26-s0001.xlsx Summary of biological, chemical, meteorological data and metadata obtained during the sampling campaign.

Data set S2 mbio.00463-26-s0002.xlsx Data and metadata for fog events characterized before, during, and after fog events.

Data set S3 mbio.00463-26-s0003.xlsx 16S rRNA sequences from single colonies isolated from fog water under enrichment with formaldehyde.

Supplemental File mbio.00463-26-s0004.pdf Supplemental figures, tables, text, and legends for Data sets S1 to S3.

ASM does not own the copyrights to Supplemental Material that may be linked to, or accessed through, an article. The authors have granted ASM a non-exclusive, world-wide license to publish the Supplemental Material files. Please contact the corresponding author directly for reuse.
