# Supplementary material for: Growth and formaldehyde degradation of photoheterotrophic Methylobacterium within radiation fogs
Source: mBio. 2026 May 11;17(6):e00463-26. doi: 10.1128/mbio.00463-26 (PMC13251464; doi:10.1128/mbio.00463-26)
Supplement: Supplemental File — Supplemental figures, tables, text, and legends for Data sets S1 to S3. [file mbio.00463-26-s0004.pdf]

Supporting Information for

Growth and formaldehyde degradation of photoheterotrophic *Methylobacterium* within radiation fogs.

Thi Thuong Thuong Cao[1, 2], Pierre Herckes[1], Derek Straub[3], Ferran Garcia-Pichel[2,4]*

1. School of Molecular Sciences; Arizona State University; Tempe, 85287 Arizona, USA

2. Center for Fundamental and Applied Microbiomics, Biodesign Institute: Arizona State University; Tempe, 85287 Arizona, USA

3. Department of Earth and Environmental Sciences; Susquehanna University, Selinsgrove, 17870 Pennsylvania, USA

4. School of Life Sciences; Arizona State University; Tempe, 85287 Arizona, USA.

* Corresponding author: Ferran Garcia-Pichel.

Email: ferran@asu.edu

This file includes:

Supporting Information Text

Figures S1 to S11

Tables S1 to S2

Legends for Datasets S1 to S3

Other supporting materials for this manuscript include the following:

Dataset S1 (separate file). Summary of biological, chemical, meteorological data and metadata obtained during the sampling campaign. Highlighted rows denote dates for which full sets of measurements before, during and after fog events were carried out.

Dataset S2 (separate file). Data and metadata for fog events characterized before, during and after fog events.

Dataset S3 (separate file). 16S rRNA sequences from single colonies isolated from fog water under enrichment with 100 mM formaldehyde as sole carbon source

Supporting Information Text

Fog water collection

Fog samples were collected with an automated Caltech Heated Rod Cloudwater Collector (CHRCC) mounted 2 m above ground having a 50% cut-off particle diameter of approximately 9 µm (1). The rods were not heated during sampling. The CHRCC draws air at 5.8 m^3 min^{-1} over a bank of 3.2 mm diameter stainless steel impaction rods where fog droplets accumulate upon impact and then flow by gravity into a sterile polyethylene bottle. A single sample was collected during each fog event. Because radiation fog formation can be difficult to predict, the operation of the CHRCC was automated to ensure reliable sample collection. A Belfort model 3100 visibility monitor was used to detect the presence of fog and to start collection by opening pneumatic doors at the CHRCC's inlet and outlet and activating the CHRCC's fan (2). A visibility threshold of 0.5 km was used to indicate the presence of dense fog. When visibility remained below 0.5 km for at least five minutes, collection was initiated and then continued until visibility exceeded this threshold for five minutes. The collector was washed with 1L of distilled water in the evenings before fog was expected to form. After washing, an additional 100 ml of distilled water was sprayed onto the collection surfaces and used as a blank. After fog dissipation, samples were transported to the laboratory for analysis and kept at 4°C for up to three weeks before anion analysis. This sampling system operated largely during the night and early morning hours as determined by the typical timing of fogs.

Anion concentration determination

Concentrations of inorganic anions in the fog samples (and blanks) were determined by ion exchange chromatography using a Thermo Scientific Dionex Integrion system with suppressed conductivity detection. Separation was obtained with a Dionex AS18 column and KOH eluent at an isocratic flow rate of 1 ml min^{-1}.

Calculation of liquid water content

The collection rate of each fog sample C_r (g min^{-1}) was calculated based on collected sample volume (g) and sampling time (min). The liquid water content LWC (g m^{-3}) was then calculated by applying the equations following equations (1):

$LWC = -0.092C_r^2 + 0.31C_r + 0.0023$ for $0.025 \leq LWC \leq 0.175$

$LWC = 0.235C_r + 0.051$ for $LWC > 0.175$

The air volume fraction of fog droplets v_f (vol%) was calculated by dividing the LWC (mg m^{-3}) by the water density ($\rho = 10^6$ mg m^{-3}).

Particle size distribution

To determine particle size distributions through flow cytometry, one half of each filter (quartz or polyethersulfone) was resuspended in 10 ml of phosphate buffer saline (PBS) at 4°C for 12 hours. The solution was then injected into an Attune NxT flow cytometer (Thermo Fisher Scientific, MA, USA) to measure particle size distribution using the forward scatter detector with a blue (488 nm) laser light source. A series of artificial 2.00, 3.30, 5.17, 7.56, 10.1, and 16.5 μm diameter polystyrene particles (Spherotech, IL, USA) at a concentration of 10^5 ml^{-1} were used as size standards. Size range gates were generated by matching gate extrema to the median intensity of the forward scatter (FSC) signal for each bead size. Data is shown as the percentage of total events whose intensities fall into each discrete size range using the FlowJo™ platform version 10.

DNA extraction

Samples for DNA extraction were processed immediately after collection. DNA extraction used the PowerSoil DNA Extraction Kit (MoBio/QIAGEN, Germany) after the method by Mazar et al.(3) with several modifications. Half of the polyethersulfone filter from the fog sample was used for DNA extraction and was cut into 0.25 cm^2 pieces with a sterile surgical scalpel. Pieces were then rolled

with sterile forceps and inserted into three PowerBead Pro Tubes with 800 µl of CD1 solution, 250 µl of phosphate buffer saline (PBS), and 150 µl of phenol: chloroform: isoamyl solution (25:24:1). The mixture was homogenized using a Mini-Beadbeater-16 (Biospec) with intervals of 30 sec of bead-beating and cooling on ice until filters were shredded. DNA extraction then followed the manufacturer's protocol, and three independent extracts were combined in one MB Spin Column at the DNA binding step. After washing the column per protocol, 500 µl of 100% ice-cold EtOH was used to remove residues. Finally, DNA was eluted and stored at –20ºC until downstream analysis. This same protocol was used in the quartz fiber filter from aerosol samples. We also processed in the same manner blank samples of distilled water with which we washed the fog sampling devices, to assess potentially contaminant DNA, from samplers or handling.

Bioinformatic analyses

High-throughput sequencing was performed on the Illumina platform with the MiSeq instrument by Microbiome Analysis Laboratory at Arizona State University (AZ, USA), yielding raw FASTQ sequence files. The sequences were analyzed via QIIME2 (v2021.11) (4) using the DADA2 plugin (5) to create a feature table containing representative sequences (ASVs) and their frequency of occurrence. The lowest number of sequence reads in any one sample was 3,450, and the highest was 152,049 reads. Resulting ASVs were then aligned against the SILVA database core reference alignment (6, 7) for taxonomic assignment. For further analysis we kept only ASVs assigned to bacteria, discarding those assigned to eukaryotic organelles (chloroplasts or mitochondria). Then, the relative abundance of a bacterial taxon was defined as the number of reads assigned to the taxon divided by the total number of reads per sample. To conduct comparative alpha and beta diversity analyses using a common denominator of sampling effort, we trimmed the reads of each sample to 4,942, which allowed us to keep most samples (one that had only 3,450 reads was discarded), while maintaining a good level of diversity saturation (90% of Shannon Diversity saturation), and without relevant effects on community diversity assessment. For the subset of 6 fog events for which we had complete, paired determinations of all types (pre-fog aerosol, fog

droplets, interstitial aerosol particles in fog, and post-fog aerosol), samples were trimmed at the depth of the lowest sample (4,638 reads) for equal effort in diversity analysis. Alpha and beta diversity metrics were computed using the diversity plugin available on QIIME2. We used Principal Coordinates Analysis (PCoA) analysis of the Bray–Curtis and Jaccard distance matrix from QIIME2 (plotted on the R vegan package (8)) for analyses of community composition similarity. To determine which specific ASVs were differentially abundant in fog and interstitial aerosol particles communities, we used the DeSeq2 method on the R BiocManager package (8). Any ASV detected as significantly different that were also detected in the blank wash water were discarded as potential contaminants (9), but this had to be done only for a few ASV, which were extremely rare in abundance.

Data availability

The sequencing data generated in this study have been deposited in the NCBI database under BioProject PRJNA1078838. Wind data was collected from Pennsylvania automated Surface Observing Systems (ASOS) network at Selinsgrove station (lat 40.82056 long -76.86389), which is 3.9 km away from the sampling site. Data can be downloaded from https://mesonet.agron.iastate.edu/request/download.phtml?network=PA_ASOS. Solar irradiance data was downloaded from National Solar Radiation Database (NSRDB), which is available on https://nsrdb.nrel.gov/data-viewer.

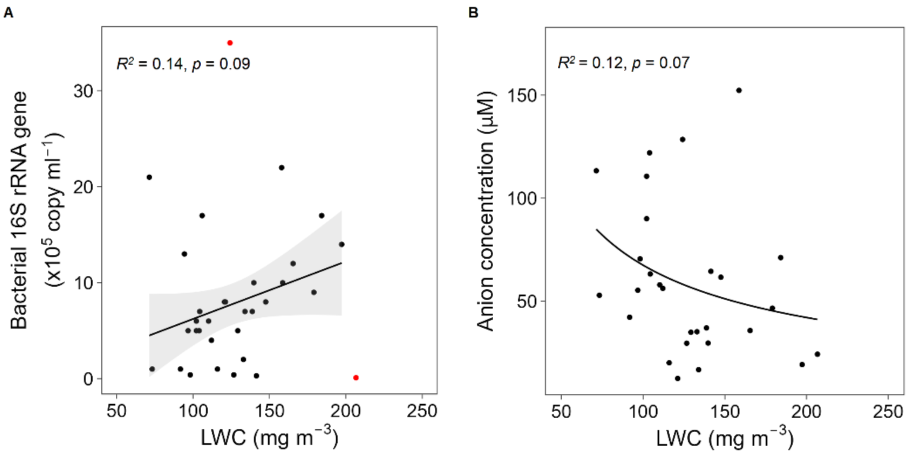

Fig. S1. Relationship between the bacterial (A) and anion (B) content in fog water as a function of liquid water content (LWC). The shaded area represents the 95% confidence interval of the linear regression, outliers are in red. See text for statistics.

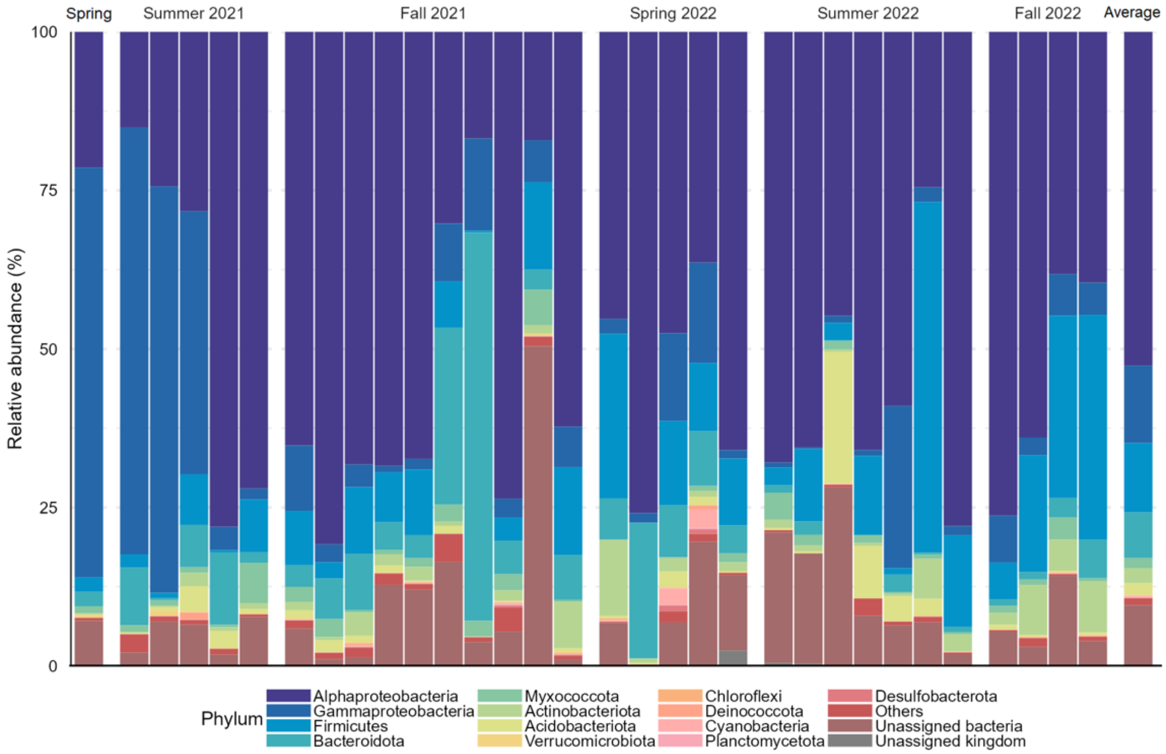

Fig. S2. Community composition of the fog water microbiome through the sampling campaign. Bacterial community composition in radiation fog from 32 sequential events and its seasonal dynamics as determined by high throughput 16S rRNA gene sequencing with phylogenetic assignments using the SILVA database and carried out to the level of phylum.

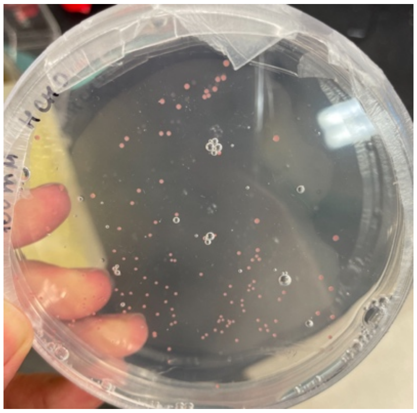

Fig. S3. *Methylobacterium*-specific viable colony count in fog water. Fifty microliters of fog water were spread on agar medium containing 1 mM formaldehyde as sole carbon source and a mineral salt medium mimicking the composition of fog water. Single-colony PCR of 4 randomly selected colonies followed by sequencing of the 16S rRNA gene confirmed that all matched with 99% identity various strains of *Methylobacterium* (see Dataset S3).

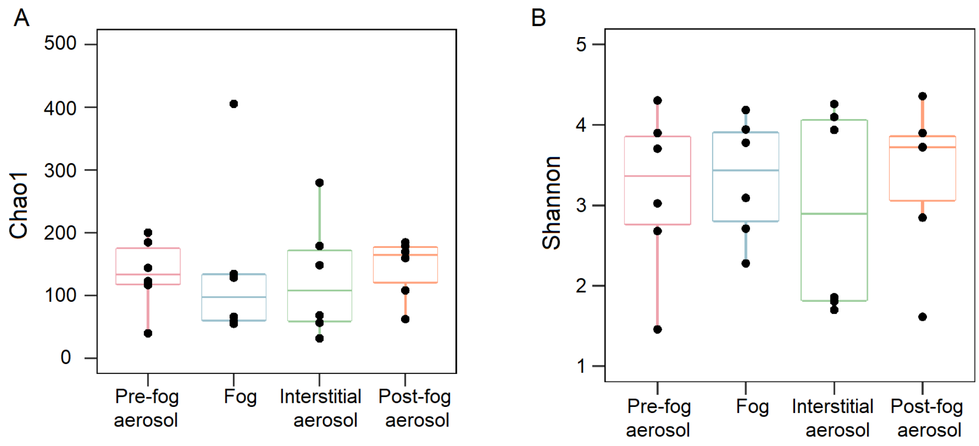

Fig. S4. Alpha diversity indices. Chao1 (A) and Shannon (B) indices for aerobiome before, during and after fog events, based on relative abundance of ASVs. Wilcoxon signed-rank tests gave no significant difference by type ($p > 0.05$) in either index. The dots on the boxplots represent the data distribution, the bars indicate quartiles 1 and 4, the box indicates quartiles 2 and 3, the horizontal line indicates the median, and the cross indicates the average.

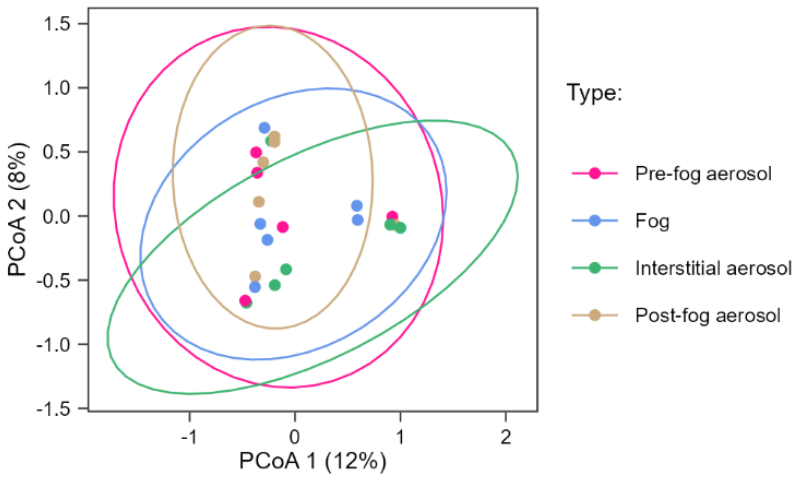

Fig. S5. PCoA separation of microbial communities by sample type based on Jaccard dissimilarity indices. Ellipses, color coded to match sample type, indicate the 95% confidence area.

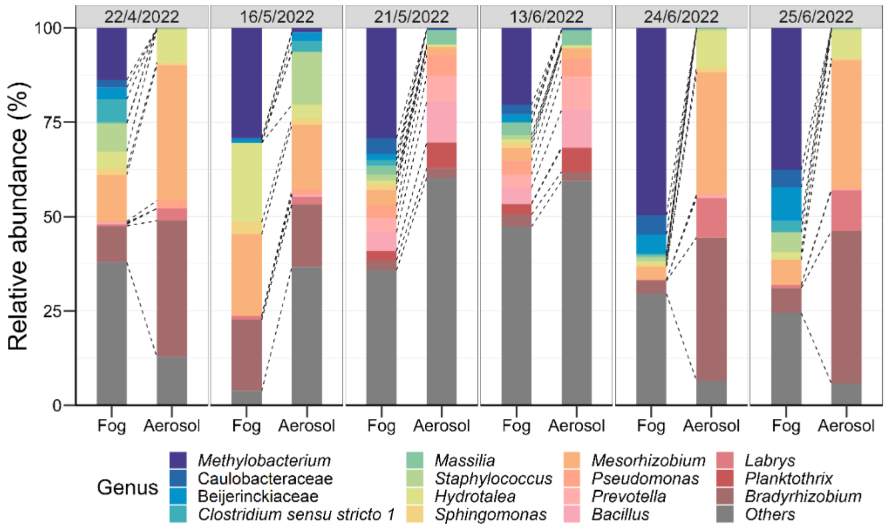

Fig. S6. Microbial composition (16S rRNA ASVs,) in fog droplets vs concurrent interstitial aerosols. Phylogenetic assignments were based on blast to the SILVA database and carried to the Genus level (including two undefined genera in the families Caulobacteraceae and Beijerinckaceae).

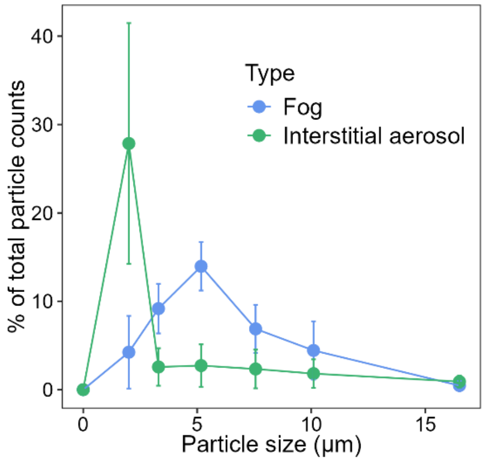

Fig. S7. Relative size distribution of particles in the fog water (fog) and interstitial aerosol particles as determined by flow cytometry of resuspended particulates, averaged from 6 independent fog events.

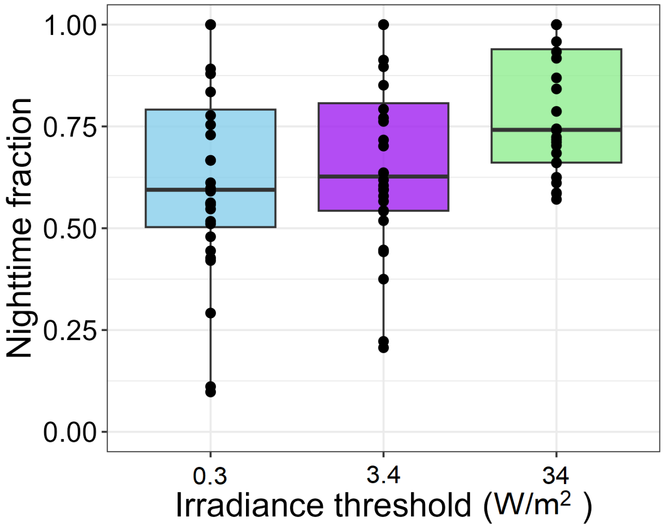

Fig. S8. Fraction of fog time without exposure to morning light during our fog sampling campaign. Data for individual fog events has been averaged (horizontal bar) for three light intensity threshold scenarios to define light vs. dark conditions.

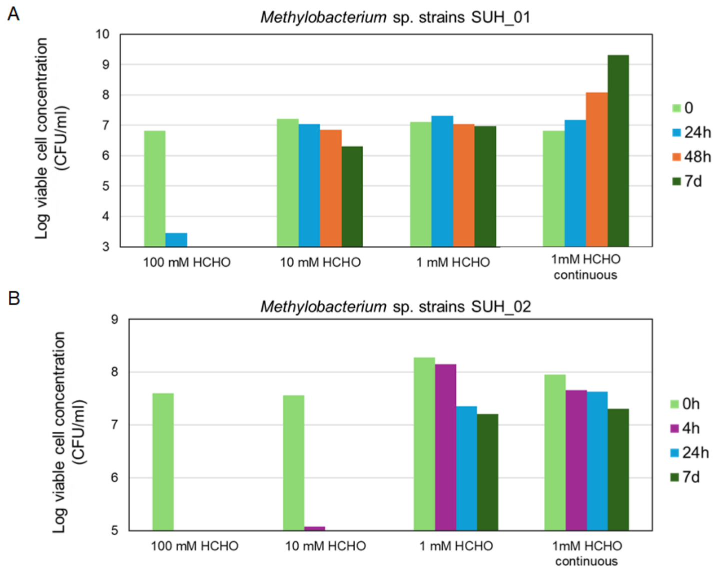

Fig. S9. Viability of *Methylobacterium* sp. strains after exposure to different HCHO concentrations. Each bar represents the average number of colony-forming units (CFU) on R2A plates for triplicate measurements, with a standard deviation of less than 5%.

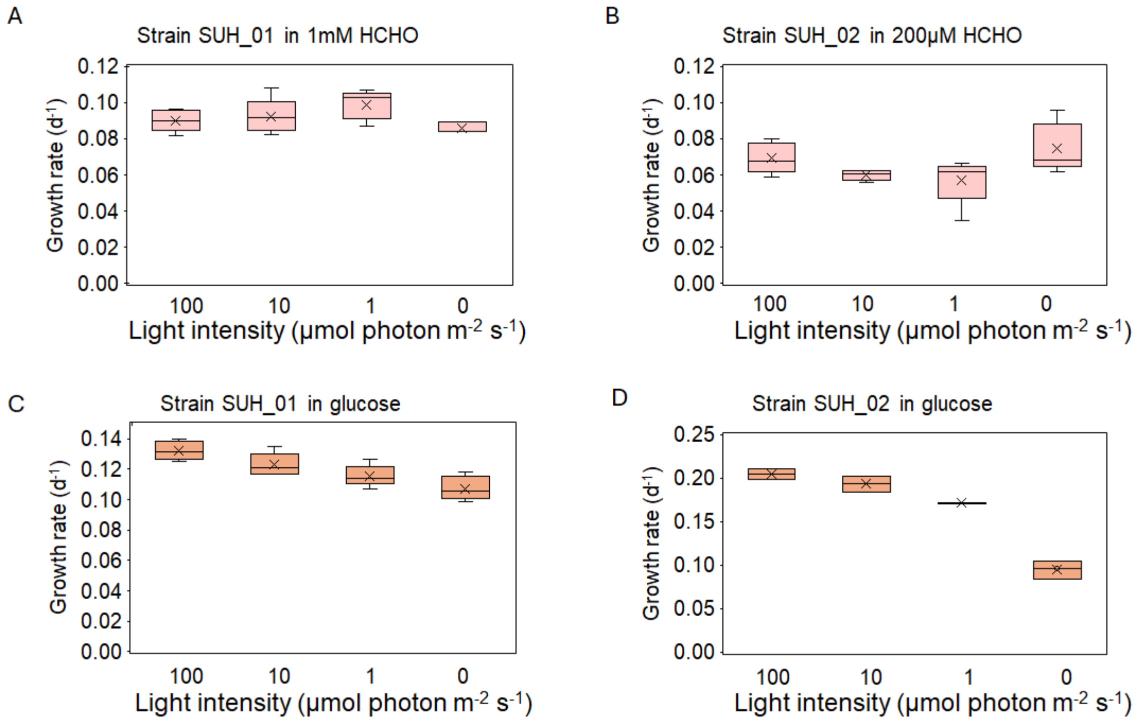

Fig. S10. Heterotrophic and photoheterotrophic growth rates of *Methylobacterium* strains as a function of carbon source and light intensity.

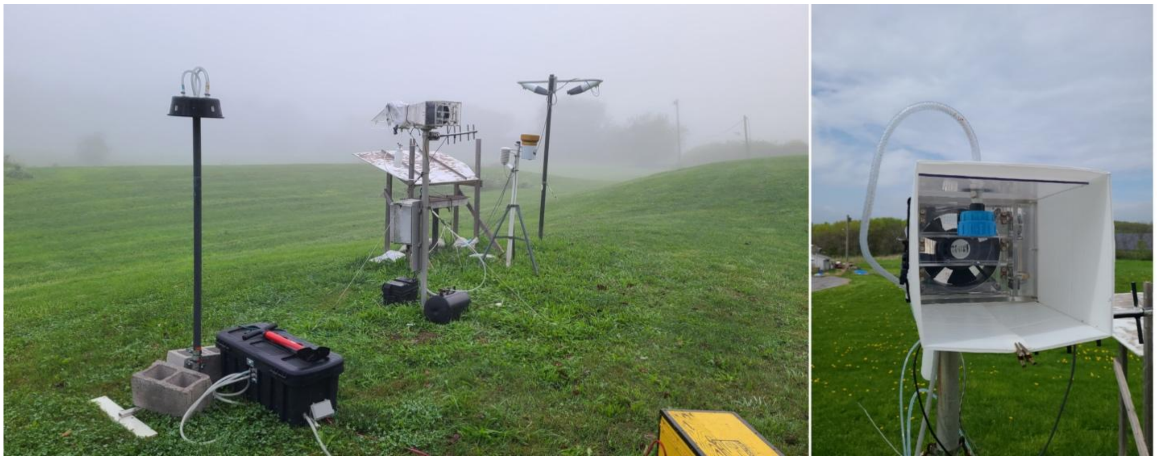

Fig. S11. Sampling devices. Left: Fog and aerosol collection set-up as installed. The ambient aerosol stand (for pre-fog and post-fog aerosol sampling) with a black circular rain shield pan is to the fore, the fog collector is in the center, and the visibility sensor is furthest away. Right: Closeup of the fog collector with an interstitial aerosol sampler (blue filter pack).

Table S1. Data and metadata from incubation experiments in Figure 5A.

Sampling Date (dd/mm/yyyy)	Sampling duration (hour)	Temperature of fog event (°C)	Sample mass (g)	Collection rate (g min^{-1})	Counted bacteria ($x10^5$ cell mL^{-1})	Initial HCHO concentration (μM)	Sampe volume for biodegradation test (untreated/killed/filtered)[a] (ml)
21/09/2022	1.5	11.9	288.2	3.2	1.5	6.3	50
6/10/2022	2.3	5.8	197.1	1.43	1.6	24	70[b]
11/10/2022	4.2	4.4	420.7	1.67	0.8	17	70
14/10/2022	6	8.3	504.3	1.4	1.6	27	100

a: Equal volume of fresh fog water (untreated) or control samples were used

b: Killed control was not performed

Table S2. Formaldehyde biodegradation rates of *Methylobacterium* strains after 7 days of incubation in mineral salt medium at pH 7.

Strain	Initial [HCHO] (mM)	% HCHO degradation	Biodegradation rate (mol $cell^{-1}s^{-1}$)
SUH_01	10	68	3.8 (± 2.6) × 10^{-18}
	1	100	9.6 (± 0.5) × 10^{-18}
	0.1	100	2.5 (± 0.3) × 10^{-19}
SUH_02	10	0	-
	1	100	2.5 (± 1.5) × 10^{-18}
	0.1	100	3.7 (± 0.3) × 10^{-18}

References

1. Demoz BB, Collett JL, Daube BC. 1996. On the Caltech Active Strand Cloudwater Collectors. Atmospheric Research 41:47-62.

2. Straub DJ. 2017. Radiation fog chemical composition and its temporal trend over an eight year period. Atmospheric Environment 148:49-61.

3. Mazar Y, Cytryn E, Erel Y, Rudich Y. 2016. Effect of Dust Storms on the Atmospheric Microbiome in the Eastern Mediterranean. Environmental Science and Technology 50:4194-4202.

4. Bolyen E, Rideout JR, Dillon MR, Bokulich NA, Abnet CC, Al-Ghalith GA, Alexander H, Alm EJ, Arumugam M, Asnicar F, Bai Y, Bisanz JE, Bittinger K, Brejnrod A, Brislawn CJ, Brown CT, Callahan BJ, Caraballo-Rodriguez AM, Chase J, ..., Caporaso JG. 2019. Reproducible, interactive, scalable and extensible microbiome data science using QIIME 2. Nat Biotechnol 37:852-857.

5. Callahan BJ, McMurdie PJ, Rosen MJ, Han AW, Johnson AJA, Holmes SP. 2016. DADA2: High-resolution sample inference from Illumina amplicon data. Nature Methods 13.

6. Pruesse E, Quast C, Knittel K, Fuchs BM, Ludwig W, Peplies J, Glöckner FO. 2007. SILVA: A comprehensive online resource for quality checked and aligned ribosomal RNA sequence data compatible with ARB. Nucleic Acids Research 35.

7. Quast C, Pruesse E, Yilmaz P, Gerken J, Schweer T, Yarza P, Peplies J, Glöckner FO. 2013. The SILVA ribosomal RNA gene database project: Improved data processing and web-based tools. Nucleic Acids Research 41.

8. Oksanen J, Blanchet FG, Kindt R, Legendre P, Minchin PR, O’hara R, Simpson GL, Solymos P, Stevens MHH, Wagner H. 2013. Community ecology package. R package version 2:321-326.

9. Couradeau E, Giraldo-Silva A, De Martini F, Garcia-Pichel F. 2019. Spatial segregation of the biological soil crust microbiome around its foundational cyanobacterium, Microcoleus vaginatus, and the formation of a nitrogen-fixing cyanosphere. Microbiome 7.
